# Supplementary figures and images for: In silico analysis and experimental validation shows negative correlation between miR-1183 and cell cycle progression gene 1 expression in colorectal cancer
Source: PLoS One. 2023 Aug 4;18(8):e0289082. doi: 10.1371/journal.pone.0289082 (PMC10403070; doi:10.1371/journal.pone.0289082)

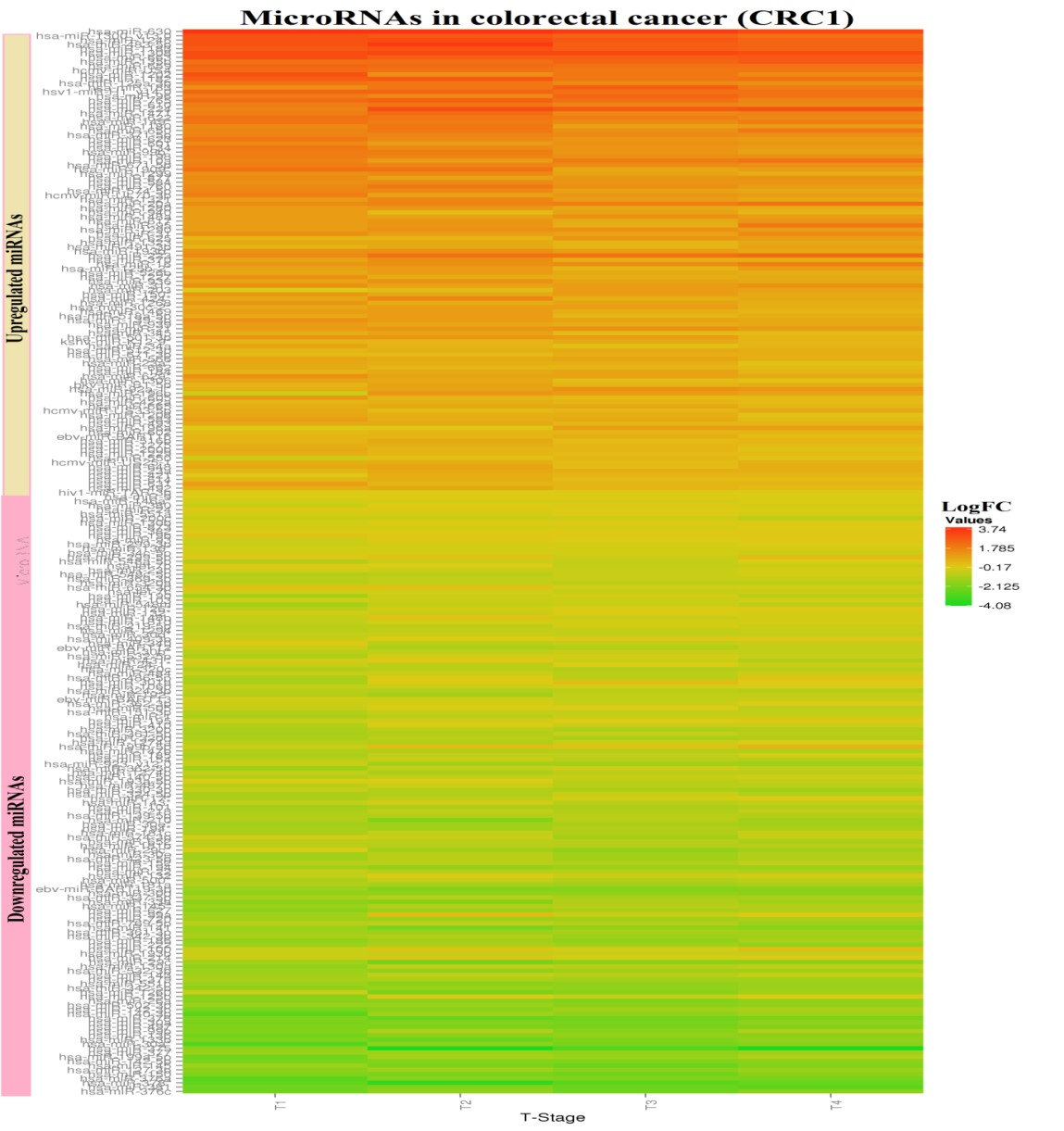

Supplement: S1 Fig — (TIF) [file pone.0289082.s001.tif]

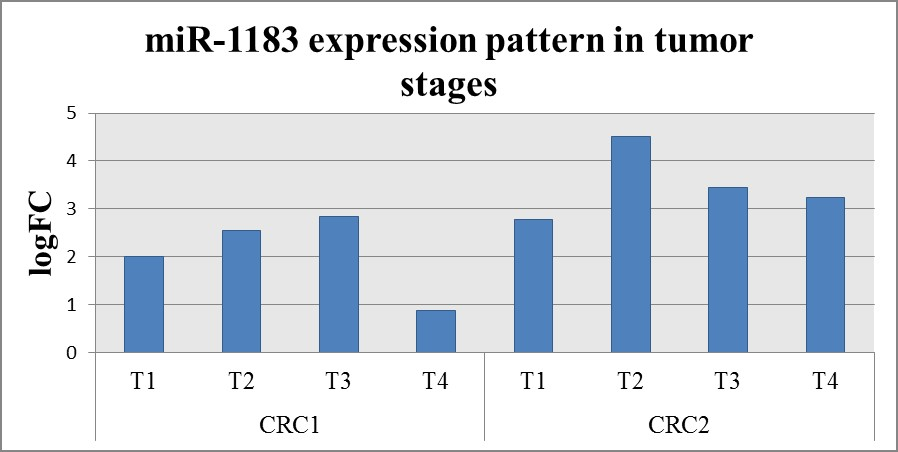

Supplement: S2 Fig — (TIF) [file pone.0289082.s002.tif]

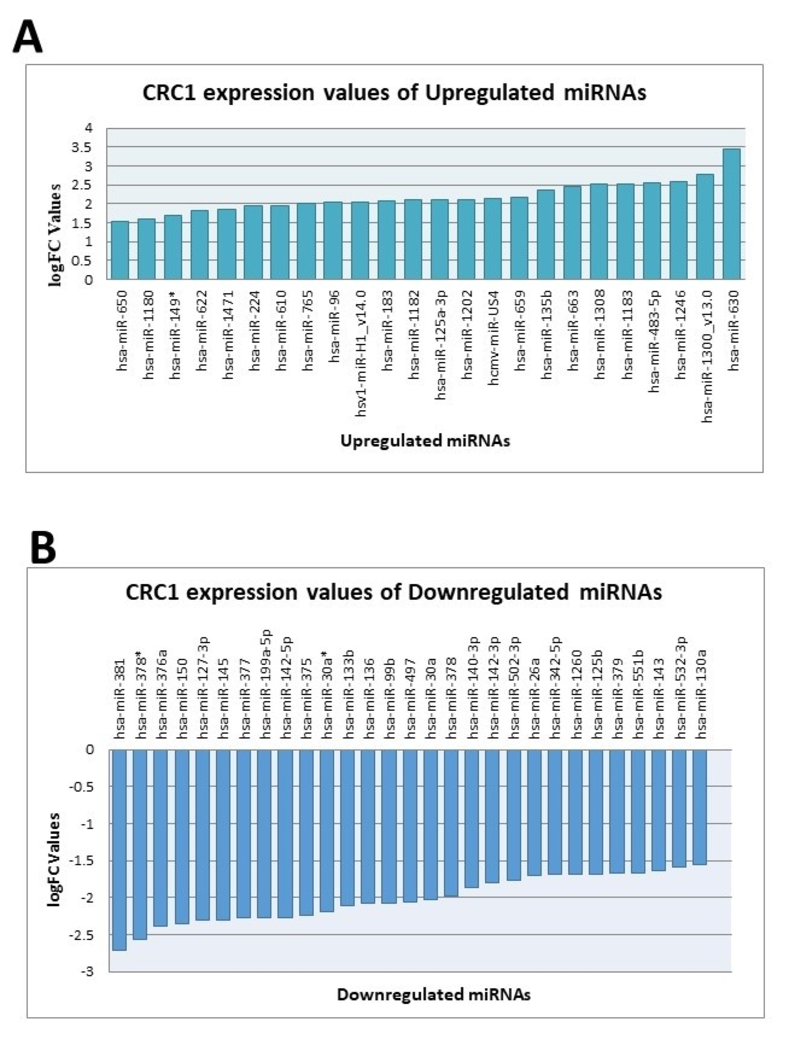

Supplement: S3 Fig — (TIF) [file pone.0289082.s003.tif]
