# Supplementary material for: In silico analysis and experimental validation shows negative correlation between miR-1183 and cell cycle progression gene 1 expression in colorectal cancer
Source: PLoS One. 2023 Aug 4;18(8):e0289082. doi: 10.1371/journal.pone.0289082 (PMC10403070; doi:10.1371/journal.pone.0289082)
Supplement: S1 File — (DOCX) [file pone.0289082.s004.docx]

**Supplementary table 1. Information of datasets used in this study.**

| Dataset | Accession Number | Title | Sample size | Platform used |
| --- | --- | --- | --- | --- |
| CRC1 | GSE41655 | MicroRNA expression profiling of colorectal normal mucosa, adenoma and adenocarcinoma tissues | 107 | Agilent-021827 Human miRNA Microarray [miRNA_107_Sep09_2_105] |
| CRC2 | GSE30454 | Colorectal cancers with microsatellite instability display unique miRNA profiles | 74 | Illumina Human v2 MicroRNA expression beadchip |
| CRC3 | GSE39814 | Profile analysis of endogenous and exosomal miRNAs in human colon cancer cell lines 1 | 19 | Agilent-021827 Human miRNA Microarray G4470C (Feature Number version) |
| CRC4 | GSE106817 | Integrated extracellular microRNA profiling for ovarian cancer screening | 2874 | 3D-Gene Human miRNA V21_1.0.0 |
| GC | GSE106817 | Integrated extracellular microRNA profiling for ovarian cancer screening | 2876 | Illumina Human v2 MicroRNA expression beadchip |
| HCC | GSE106817 | Integrated extracellular microRNA profiling for ovarian cancer screening | 2842 | Illumina Human v2 MicroRNA expression beadchip |

**Supplementary table 2. Oligos used in this study.**

| # | Name of oligo | Seq (5’ – 3’) |
| --- | --- | --- |
| 1 | miR-1183 (SL) | GAAAGAAGGCGAGGAGCAGATCGAGGAAGAAGACGGAAGAATGTGCGTCTCGCCTTCTTTCCGATCACC |
| 2 | miR-1183 (F) | GCGCCACTGTAGGTGATCG |
| 3 | miR-1183 (R) | CCAGTGCAGGGTCCGAGGTA |
| 4 | CCPG1 (F) | TAGTAGAGGAGCAGTGGCG |
| 5 | CCPG1 (R) | TGTTCGGTATAGCACTCGGG |
| 6 | U6 (F) | CGATAAAATTGGAACGATACAG |
| 7 | U6 (R) | TCGATTTGTGCGTGTCATCCT |

**Supplementary table 3. Clinical features of CRC1 dataset.**

| Characteristics | Colorectal adenocarcinoma | Colorectal adenoma | Colorectal  Normal mucosa |
| --- | --- | --- | --- |
| Sample size (n) | 33 | 59 | 15 |
| Gender |  |  |  |
| Male | 24 | 38 | 8 |
| Female | 9 | 21 | 7 |
| Age |  |  |  |
| Below 60 yrs. | 9 | 27 | 10 |
| 60 yrs. | 1 | 1 | 1 |
| Above 60 yrs. | 23 | 31 | 4 |
| High grade dysplasia |  | 20 |  |
| Low grade dysplasia |  | 39 |  |
| T- Stage |  |  |  |
| T1 | 6 |  |  |
| T2 | 8 |  |  |
| T3 | 11 |  |  |
| T4 | 8 |  |  |
| N-stage |  |  |  |
| 0 | 15 |  |  |
| I | 15 |  |  |
| II | 3 |  |  |
| M-stage |  |  |  |
| 0 | 31 |  |  |
| I | 2 |  |  |

**Supplementary table 4. Differentially expressed miRNAs in CRC1 (p**≤**0.05)**

| miRNA names | P-value | logFC values |  |
| --- | --- | --- | --- |
| UPREGULATED miRNAs |  |  |  |
| hsa-miR-630 | 4.98E-12 | 3.45 |  |
| hsa-miR-1300_v13.0 | 2.32E-10 | 2.76 |  |
| hsa-miR-1246 | 8.28E-10 | 2.57 |  |
| hsa-miR-483-5p | 2.91E-08 | 2.56 |  |
| hsa-miR-1183 | 3.30E-08 | 2.52 |  |
| hsa-miR-1308 | 2.67E-14 | 2.51 |  |
| hsa-miR-663 | 2.74E-09 | 2.45 |  |
| hsa-miR-135b | 2.18E-09 | 2.37 |  |
| hsa-miR-659 | 1.38E-06 | 2.17 |  |
| hcmv-miR-US4 | 9.36E-07 | 2.13 |  |
| hsa-miR-1202 | 6.53E-06 | 2.12 |  |
| hsa-miR-1182 | 3.09E-08 | 2.1 |  |
| hsa-miR-125a-3p | 8.39E-05 | 2.1 |  |
| hsa-miR-183 | 1.65E-07 | 2.06 |  |
| hsv1-miR-H1_v14.0 | 3.31E-06 | 2.05 |  |
| hsa-miR-96 | 2.92E-07 | 2.04 |  |
| hsa-miR-765 | 5.08E-07 | 2.02 |  |
| hsa-miR-610 | 4.65E-07 | 1.96 |  |
| hsa-miR-224 | 6.82E-07 | 1.95 |  |
| hsa-miR-1471 | 2.25E-05 | 1.85 |  |
| hsa-miR-622 | 3.59E-05 | 1.82 |  |
| hsa-miR-149* | 2.19E-05 | 1.7 |  |
| hsa-miR-1180 | 2.32E-04 | 1.61 |  |
| hsa-miR-650 | 2.28E-04 | 1.54 |  |
| hsa-miR-371-5p | 8.41E-05 | 1.49 |  |
| hsa-miR-623 | 3.40E-05 | 1.47 |  |
| hsa-miR-601 | 2.57E-04 | 1.43 |  |
| hsa-miR-134 | 9.90E-03 | 1.41 |  |
| hsa-miR-99b* | 3.23E-03 | 1.4 |  |
| hsa-miR-198 | 6.42E-06 | 1.39 |  |
| hsa-miR-10a | 7.66E-04 | 1.37 |  |
| hsa-miR-671-5p | 1.90E-03 | 1.33 |  |
| hsa-miR-1909* | 5.56E-04 | 1.28 |  |
| hsa-miR-1299 | 2.08E-04 | 1.26 |  |
| hsa-miR-877 | 9.77E-05 | 1.25 |  |
| hsa-miR-584 | 3.85E-05 | 1.22 |  |
| hsa-miR-760 | 6.00E-04 | 1.2 |  |
| hsa-miR-574-5p | 1.67E-03 | 1.19 |  |
| hcmv-miR-UL70-3p | 1.18E-02 | 1.16 |  |
| hsa-miR-1321 | 5.27E-06 | 1.15 |  |
| hsa-miR-20a | 3.67E-03 | 1.13 |  |
| hsa-miR-1280 | 2.34E-07 | 1.1 |  |
| hsa-miR-940 | 3.35E-04 | 1.04 |  |
| hsa-miR-148a | 1.72E-04 | 1.03 |  |
| hsa-miR-617 | 3.01E-04 | 1.03 |  |
| hsa-miR-95 | 2.80E-02 | 1.02 |  |
| hsa-miR-1290 | 6.40E-04 | 1 |  |
| hsa-miR-31 | 2.08E-02 | 1 |  |
| hsa-miR-625 | 5.76E-03 | 9.98E-01 |  |
| hsa-miR-1323 | 9.40E-03 | 9.95E-01 |  |
| hsa-miR-491-3p | 4.47E-03 | 9.77E-01 |  |
| hsa-miR-193b* | 2.01E-03 | 9.59E-01 |  |
| hsa-miR-223 | 9.04E-03 | 9.53E-01 |  |
| hsa-miR-370 | 7.19E-03 | 9.42E-01 |  |
| hsa-miR-16 | 2.45E-02 | 9.33E-01 |  |
| hsa-miR-125b-2* | 6.84E-03 | 9.29E-01 |  |
| hsa-miR-526b | 5.90E-04 | 9.28E-01 |  |
| hsa-miR-1227 | 4.80E-04 | 9.10E-01 |  |
| hsa-miR-936 | 4.49E-03 | 8.93E-01 |  |
| hsa-miR-31* | 3.43E-02 | 8.78E-01 |  |
| hsa-miR-203 | 2.91E-02 | 8.52E-01 |  |
| hsa-miR-150* | 1.52E-02 | 8.30E-01 |  |
| hsa-miR-424* | 1.40E-02 | 8.08E-01 |  |
| hsa-miR-1268 | 1.10E-02 | 8.07E-01 |  |
| hsa-miR-30c-2* | 4.50E-03 | 7.93E-01 |  |
| hsa-miR-1469 | 7.72E-03 | 7.91E-01 |  |
| hsa-miR-518a-5p | 7.63E-03 | 7.76E-01 |  |
| hsa-miR-139-3p | 1.85E-02 | 7.70E-01 |  |
| dmr_285 | 2.71E-03 | 7.69E-01 |  |
| hsa-miR-939 | 1.37E-02 | 7.67E-01 |  |
| hsa-miR-21 | 2.10E-03 | 7.39E-01 |  |
| hsa-miR-345 | 4.84E-02 | 7.34E-01 |  |
| hsa-miR-501-3p | 7.73E-03 | 7.30E-01 |  |
| kshv-miR-K12-9* | 1.08E-02 | 7.03E-01 |  |
| hsa-miR-34a | 2.95E-04 | 6.82E-01 |  |
| hsa-miR-512-3p | 4.95E-03 | 6.79E-01 |  |
| hsa-miR-671-3p | 2.24E-03 | 6.77E-01 |  |
| hsa-miR-566 | 6.95E-03 | 6.64E-01 |  |
| hsa-miR-23a* | 4.28E-02 | 6.57E-01 |  |
| hsa-miR-662 | 2.15E-02 | 6.51E-01 |  |
| hsa-miR-184 | 4.07E-03 | 6.45E-01 |  |
| hsa-miR-629* | 2.81E-02 | 6.44E-01 |  |
| hsa-miR-1306 | 5.93E-03 | 6.40E-01 |  |
| bkv-miR-B1-5p | 1.89E-02 | 6.40E-01 |  |
| hsa-miR-92a-1* | 2.06E-02 | 6.31E-01 |  |
| hsa-miR-196b | 3.81E-02 | 6.26E-01 |  |
| hsa-miR-605 | 3.79E-02 | 6.21E-01 |  |
| hsa-miR-422a | 3.35E-02 | 6.19E-01 |  |
| hsa-miR-665 | 4.49E-02 | 6.19E-01 |  |
| hcmv-miR-US33-5p | 2.80E-02 | 6.15E-01 |  |
| hsa-miR-1208 | 1.00E-02 | 5.80E-01 |  |
| hsa-miR-583 | 1.79E-02 | 5.78E-01 |  |
| hsa-miR-493 | 1.65E-02 | 5.77E-01 |  |
| hsa-miR-196a | 3.07E-02 | 5.75E-01 |  |
| hsa-miR-602 | 1.60E-04 | 5.66E-01 |  |
| hur_4 | 1.08E-02 | 5.39E-01 |  |
| ebv-miR-BART16 | 4.32E-02 | 5.30E-01 |  |
| hsa-miR-516b | 5.48E-03 | 5.28E-01 |  |
| hsa-miR-1275 | 2.41E-02 | 5.19E-01 |  |
| hsa-miR-200b | 2.75E-02 | 5.19E-01 |  |
| hsa-miR-1229 | 4.58E-02 | 5.12E-01 |  |
| hsa-miR-550 | 1.83E-02 | 5.05E-01 |  |
| hcmv-miR-US25-1 | 1.93E-02 | 5.05E-01 |  |
| hsa-miR-648 | 9.02E-03 | 5.01E-01 |  |
| hsa-miR-23a | 1.02E-03 | 4.49E-01 |  |
| hsa-miR-421 | 3.51E-02 | 4.47E-01 |  |
| hsa-miR-614 | 1.01E-02 | 3.97E-01 |  |
| hsa-miR-631 | 4.27E-02 | 3.86E-01 |  |
| hsa-miR-492 | 4.32E-02 | 3.81E-01 |  |
| DOWNREGULATED miRNAs |  |  |  |
| hiv1-miR-TAR-3p | 3.36E-02 | -1.64E-01 | |
| hsa-miR-9 | 4.68E-02 | -2.18E-01 | |
| hur_1 | 2.69E-02 | -2.74E-01 | |
| hsa-miR-146a* | 2.89E-03 | -2.92E-01 | |
| hsa-miR-380 | 4.21E-02 | -2.96E-01 | |
| hsa-miR-24 | 2.40E-02 | -3.00E-01 | |
| hsa-miR-551a | 2.74E-02 | -3.04E-01 | |
| hsa-miR-200c | 3.77E-02 | -3.18E-01 | |
| hsa-miR-130b | 3.56E-02 | -3.27E-01 | |
| hsa-miR-873 | 1.16E-02 | -3.42E-01 | |
| hsa-miR-382 | 2.63E-02 | -3.51E-01 | |
| hsa-miR-19b | 3.56E-02 | -3.65E-01 | |
| hsa-miR-93 | 4.59E-02 | -3.79E-01 | |
| hsa-miR-299-3p | 8.92E-04 | -4.00E-01 | |
| hsa-miR-136* | 1.88E-07 | -4.01E-01 | |
| hsa-miR-34c-5p | 3.55E-03 | -4.16E-01 | |
| hsa-miR-299-5p | 1.27E-02 | -4.45E-01 | |
| hsa-miR-548a-5p | 1.91E-02 | -4.55E-01 | |
| hsa-let-7b | 1.40E-03 | -4.82E-01 | |
| hsa-miR-23b | 2.51E-02 | -4.83E-01 | |
| hsa-miR-548c-5p | 4.32E-02 | -4.90E-01 | |
| hsa-miR-369-3p | 8.71E-03 | -4.92E-01 | |
| hsa-miR-320a | 4.03E-03 | -5.16E-01 | |
| hsa-miR-654-3p | 6.54E-03 | -5.19E-01 | |
| hsa-let-7c | 1.88E-03 | -5.21E-01 | |
| hsa-miR-190 | 4.40E-02 | -5.26E-01 | |
| hsa-miR-103 | 3.29E-02 | -5.29E-01 | |
| hsa-miR-548m | 3.85E-02 | -5.52E-01 | |
| hsa-miR-126* | 9.69E-03 | -5.58E-01 | |
| hsa-miR-132* | 3.27E-03 | -5.64E-01 | |
| hsa-miR-148b | 3.45E-02 | -5.75E-01 | |
| hsa-miR-181d | 9.25E-03 | -5.82E-01 | |
| hsa-miR-219-5p | 2.01E-04 | -5.90E-01 | |
| hsa-miR-1294 | 1.42E-03 | -5.90E-01 | |
| hsa-miR-30d* | 1.75E-03 | -5.91E-01 | |
| hsa-miR-409-3p | 1.64E-02 | -6.06E-01 | |
| hsa-miR-340 | 1.11E-04 | -6.17E-01 | |
| ebv-miR-BART12 | 1.81E-02 | -6.39E-01 | |
| hsa-miR-30b* | 2.41E-03 | -6.44E-01 | |
| hsa-miR-532-5p | 9.60E-04 | -6.58E-01 | |
| hsa-miR-431* | 3.43E-02 | -6.65E-01 | |
| hsa-miR-24-1* | 4.17E-02 | -6.70E-01 | |
| hsa-miR-320c | 1.80E-05 | -6.74E-01 | |
| hsa-miR-484 | 2.54E-03 | -6.89E-01 | |
| hsa-miR-486-5p | 2.24E-02 | -7.01E-01 | |
| hsa-miR-301a | 4.00E-02 | -7.09E-01 | |
| hsa-miR-106b | 1.05E-03 | -7.19E-01 | |
| hsa-miR-324-3p | 2.36E-06 | -7.29E-01 | |
| hsa-miR-192* | 3.07E-02 | -7.37E-01 | |
| ebv-miR-BART13 | 3.61E-02 | -7.39E-01 | |
| hsa-miR-362-3p | 1.65E-02 | -7.40E-01 | |
| hsa-miR-505 | 1.05E-03 | -7.89E-01 | |
| hsa-miR-151-3p | 5.31E-06 | -8.00E-01 | |
| hsa-miR-1 | 9.13E-03 | -8.10E-01 | |
| hsa-miR-195 | 2.73E-02 | -8.18E-01 | |
| hsa-miR-410 | 9.84E-05 | -8.23E-01 | |
| hsa-miR-320b | 8.21E-08 | -8.24E-01 | |
| hsa-miR-361-5p | 6.19E-06 | -8.27E-01 | |
| hsa-miR-320d | 3.16E-08 | -8.38E-01 | |
| hsa-miR-1274a | 3.50E-03 | -8.38E-01 | |
| hsa-miR-199b-5p | 1.50E-02 | -8.79E-01 | |
| hsa-miR-147b | 1.98E-07 | -8.94E-01 | |
| hsa-miR-185 | 3.94E-05 | -9.09E-01 | |
| hsa-miR-154 | 1.13E-06 | -9.13E-01 | |
| hsa-miR-923_v12.0 | 9.30E-03 | -9.20E-01 | |
| hsa-miR-362-5p | 1.34E-03 | -9.24E-01 | |
| hsa-miR-1274b | 7.67E-06 | -9.34E-01 | |
| hsa-miR-140-5p | 4.24E-04 | -9.41E-01 | |
| hsa-miR-193a-5p | 1.76E-03 | -9.41E-01 | |
| hsa-miR-487b | 1.14E-08 | -9.53E-01 | |
| hsa-miR-330-3p | 3.29E-05 | -9.53E-01 | |
| hsa-miR-324-5p | 5.11E-04 | -9.54E-01 | |
| hsa-miR-17* | 3.03E-03 | -9.54E-01 | |
| hsa-miR-143* | 3.94E-07 | -9.71E-01 | |
| hsa-miR-101 | 1.75E-05 | -9.91E-01 | |
| hsa-miR-218 | 9.80E-10 | -9.94E-01 | |
| hsa-miR-139-5p | 7.84E-10 | -1 | |
| hsa-miR-210 | 4.59E-04 | -1.01 | |
| hsa-miR-30e* | 2.65E-03 | -1.01 | |
| hsa-miR-194* | 2.79E-07 | -1.03 | |
| hsa-miR-181c | 4.44E-04 | -1.03 | |
| hsa-miR-574-3p | 9.10E-03 | -1.04 | |
| hsa-miR-652 | 4.60E-05 | -1.07 | |
| hsa-miR-181b | 5.21E-05 | -1.07 | |
| hsa-miR-29c* | 5.75E-05 | -1.11 | |
| hsa-miR-30e | 5.34E-09 | -1.12 | |
| hsa-miR-423-5p | 1.94E-05 | -1.12 | |
| hsa-miR-152 | 1.17E-11 | -1.14 | |
| hsa-miR-194 | 2.48E-09 | -1.14 | |
| hsa-miR-22 | 3.37E-10 | -1.15 | |
| hsa-miR-132 | 1.17E-03 | -1.15 | |
| hsa-miR-500* | 1.15E-05 | -1.19 | |
| hsa-miR-181a | 6.70E-07 | -1.2 | |
| ebv-miR-BART19-3p | 3.20E-03 | -1.21 | |
| hsa-miR-30d | 1.21E-10 | -1.23 | |
| hsa-miR-337-5p | 3.47E-13 | -1.27 | |
| hsa-miR-33a | 1.34E-05 | -1.27 | |
| hsa-miR-145* | 2.77E-07 | -1.28 | |
| hsa-miR-627 | 6.68E-07 | -1.33 | |
| hsa-miR-99a | 1.35E-04 | -1.34 | |
| hsa-miR-720 | 1.26E-08 | -1.35 | |
| hsa-miR-769-5p | 1.21E-06 | -1.37 | |
| hsa-miR-141 | 3.30E-09 | -1.38 | |
| hsa-miR-361-3p | 5.51E-09 | -1.4 | |
| hsa-miR-342-3p | 7.29E-12 | -1.42 | |
| hsa-miR-186 | 9.20E-12 | -1.43 | |
| hsa-miR-222 | 8.89E-05 | -1.44 | |
| hsa-miR-100 | 1.28E-03 | -1.44 | |
| hsa-miR-193b | 2.45E-05 | -1.45 | |
| hsa-miR-214 | 1.56E-04 | -1.47 | |
| hsa-miR-29c | 5.58E-11 | -1.49 | |
| hsa-miR-130a | 3.20E-09 | -1.56 | |
| hsa-miR-532-3p | 3.85E-08 | -1.58 | |
| hsa-miR-143 | 1.22E-05 | -1.63 | |
| hsa-miR-379 | 3.57E-16 | -1.66 | |
| hsa-miR-551b | 5.99E-10 | -1.66 | |
| hsa-miR-342-5p | 1.32E-12 | -1.68 | |
| hsa-miR-1260 | 8.28E-06 | -1.68 | |
| hsa-miR-125b | 1.33E-04 | -1.68 | |
| hsa-miR-26a | 6.71E-10 | -1.7 | |
| hsa-miR-502-3p | 4.29E-09 | -1.76 | |
| hsa-miR-142-3p | 4.30E-06 | -1.79 | |
| hsa-miR-140-3p | 2.44E-10 | -1.86 | |
| hsa-miR-378 | 2.56E-11 | -1.98 | |
| hsa-miR-30a | 1.30E-12 | -2.03 | |
| hsa-miR-497 | 1.29E-21 | -2.06 | |
| hsa-miR-99b | 3.57E-09 | -2.07 | |
| hsa-miR-136 | 1.20E-17 | -2.08 | |
| hsa-miR-133b | 2.01E-12 | -2.1 | |
| hsa-miR-30a* | 7.34E-21 | -2.19 | |
| hsa-miR-375 | 3.58E-07 | -2.23 | |
| hsa-miR-377 | 8.47E-14 | -2.27 | |
| hsa-miR-199a-5p | 9.68E-11 | -2.27 | |
| hsa-miR-142-5p | 2.71E-09 | -2.27 | |
| hsa-miR-145 | 1.70E-14 | -2.3 | |
| hsa-miR-127-3p | 5.47E-13 | -2.31 | |
| hsa-miR-150 | 4.48E-08 | -2.35 | |
| hsa-miR-376a | 1.30E-18 | -2.39 | |
| hsa-miR-378* | 1.25E-14 | -2.57 | |
| hsa-miR-381 | 2.75E-22 | -2.71 | |
| hsa-miR-376c | 2.60E-14 | -2.86 | |

**Supplementary table 5. List of overlapping miRNAs of colorectal cancer datasets (CRC1 with CRC2, CRC3, and CRC4)**

| CRC1 and CRC2 | CRC1 and CRC3 | CRC1 and CRC4 |
| --- | --- | --- |
| hsa-miR-630  hsa-miR-1183  hsa-miR-1182  hsa-miR-622 | **hsa-miR-630**  hsa-miR-1246  hsa-miR-483-5p  **hsa-miR-1183**  hsa-miR-663  hsa-miR-135b  hsa-miR-659  hsa-miR-1202  hsa-miR-1182  hsa-miR-183  hsa-miR-96  hsa-miR-765  hsa-miR-610  hsa-miR-224  hsa-miR-622  hsa-miR-1180  hsv1-miR-H1_v14.0 | **hsa-miR-630**  hsa-miR-1246  **hsa-miR-1183**  hsa-miR-610  hsa-miR-650 |

**Supplementary table 6. Correlation values of genes (Pearson r ≤ 0.5; p-value ≤ 0.05).**

| Full name | Gene Symbol | *Gene IDs* | *Correlation values(Pearson r)* |
| --- | --- | --- | --- |
| occludin/ELL domain containing 1(OCEL1) | OCEL1 | A_23_P90523 | -0.573676753 |
| chromosome 14 open reading frame 28(C14orf28) | c14orf28 | A_24_P33048 | -0.571173739 |
| matrix metallopeptidase 7(MMP7) | MMP7 | A_23_P52761 | -0.567359551 |
| single-pass membrane protein with coiled-coil domains 4 | SMCO4 | A_23_P75430 | -0.566978466 |
| cysteine rich transmembrane BMP regulator 1(CRIM1) | CRIM1 | A_23_P51105 | -0.566001435 |
| chitobiase(CTBS) | CTBS | A_24_P940135 | -0.559030774 |
| related RAS viral (r-ras) oncogene homolog(RRAS) | RRAS | A_23_P39076 | -0.558673277 |
| zinc finger DHHC-type containing 2(ZDHHC2) | ZDHHC2 | A_23_P9086 | -0.558573458 |
| PQ loop repeat containing 3(PQLC3) | PQLC3 | A_23_P131375 | -0.557957991 |
| TRIO and F-actin binding protein(TRIOBP) | TRIOBP | A_23_P17855 | -0.553360687 |
| yippee like 5(YPEL5) | YPEL5 | A_23_P108835 | -0.55212688 |
| LMBR1 domain containing 1(LMBRD1) | LMBRD1 | A_23_P81660 | -0.547162148 |
| hexosaminidase subunit alpha(HEXA) | HEXA | A_24_P912058 | -0.546874795 |
| 2-oxoglutarate and iron dependent oxygenase domain containing 3(OGFOD3) | OGFOD3 | A_23_P124132 | -0.543397073 |
| transmembrane protein 165(TMEM165) | TMEM165 | A_24_P43391 | -0.542830082 |
| echinoderm microtubule associated protein like 2(EML2) | EML2 | A_23_P101297 | -0.537419597 |
| baculoviral IAP repeat containing 2(BIRC2) | BIRC2 | A_24_P115774 | -0.535724185 |
| praja ring finger ubiquitin ligase 2(PJA2) | PJA2 | A_23_P133470 | -0.534959453 |
| transmembrane p24 trafficking protein 3(TMED3) | TMED3 | A_23_P26173 | -0.533658284 |
| cold inducible RNA binding protein(CIRBP) | CIRBP | A_23_P377616 | -0.530310869 |
| SWAP switching B-cell complex 70kDa subunit(SWAP70) | SWAP70 | A_23_P116533 | -0.529734541 |
| steroid 5 alpha-reductase 3(SRD5A3) | SRD5A3 | A_23_P121783 | -0.528677649 |
| transmembrane protein 50B(TMEM50B) | TMEM50B | A_23_P57304 | -0.527660436 |
| TNFSF12-TNFSF13 readthrough(TNFSF12-TNFSF13) | TNFSF12-TNFS | A_24_P245298 | -0.522607892 |
| transmembrane channel like 4(TMC4) | TMC4 | A_23_P330461 | -0.521171431 |
| melanocortin 2 receptor accessory protein 2(MRAP2) | MRAP2 | A_32_P41254 | -0.521131823 |
| ankyrin repeat and BTB domain containing 1(ABTB1) | ABTB1 | A_24_P200297 | -0.519859955 |
| arrestin domain containing 3(ARRDC3) | ARRDC3 | A_24_P274615 | -0.519324461 |
| exocyst complex component 7(EXOC7) | EXOC7 | A_23_P100556 | -0.519230952 |
| polypeptide N-acetylgalactosaminyltransferase 13(GALNT13) | GALNT13 | A_23_P306105 | -0.516121605 |
| seizure related 6 homolog like 2(SEZ6L2) | SEZ6L2 | A_23_P10194 | -0.514944332 |
| interferon alpha inducible protein 27 like 1(IFI27L1) | IFI27L1 | A_23_P53976 | -0.513606273 |
| fasciculation and elongation protein zeta 2(FEZ2) | FEZ2 | A_23_P39718 | -0.513546338 |
| FCH domain only 2(FCHO2) | FCHO2 | A_23_P349083 | -0.51109865 |
| KIAA1107(KIAA1107) | KIAA1107 | A_32_P98136 | -0.508132071 |
| proline rich nuclear receptor coactivator 1(PNRC1) | PNRC1 | A_23_P145074 | -0.506915314 |
| SH3 domain binding glutamate rich protein like(SH3BGRL) | SH3BGRL | A_23_P148297 | -0.505900328 |
| dedicator of cytokinesis 8(DOCK8) | DOCK8 | A_32_P181077 | -0.504553565 |
| cell cycle progression 1(CCPG1) | CCPG1 | A_23_P346006 | -0.503772735 |
| transmembrane protein 134(TMEM134) | TMEM134 | A_23_P161522 | -0.503761251 |
| interferon alpha inducible protein 27 like 1(IFI27L1) | IFI27L1 | A_24_P261929 | -0.503195069 |
| chromosome 11 open reading frame 52(C11orf52) | C11ORF52 | A_23_P1722 | -0.502164271 |
| transmembrane protein 136(TMEM136) | TMEM136 | A_23_P329890 | -0.501498298 |

**Supplementary table 7. Biological functions of genes common among Target scan, miRmap, and microarray expression data of CRC1 dataset.**

| Gene Name | Biological Function | Biological process |
| --- | --- | --- |
| CRIM1 | PDZ domain binding  insulin-like growth factor-activated receptor activity  serine-type endopeptidase inhibitor activity | insulin-like growth factor receptor signaling pathway  negative regulation of endopeptidase activity  nervous system development |
| PJA2  praja ring finger ubiquitin ligase 2 | metal ion binding  protein binding  protein kinase A catalytic subunit binding  ubiquitin protein ligase activity  ubiquitin-protein transferase activity | hippo signaling  inflammatory response  innate immune response  long-term memory  positive regulation of JNK cascade, p38MAPK cascade, toll-like receptor 2 signaling pathway  protein ubiquitination  regulation of macrophage activation and protein kinase A signaling |
| PNRC1  proline rich nuclear receptor coactivator 1 | protein binding |  |
| CCPG1  cell cycle progression 1 | Molecular function  protein binding | cell cycle  positive regulation of cell cycle  positive regulation of cell proliferation  positive regulation of transcription by RNA polymerase II  regulation of Rho guanyl-nucleotide exchange factor activity |
| TMEM136  transmembrane protein 136 | Molecular function | Biological process |
