# Supplementary material for: In silico analysis and experimental validation shows negative correlation between miR-1183 and cell cycle progression gene 1 expression in colorectal cancer
Source: PLoS One. 2023 Aug 4;18(8):e0289082. doi: 10.1371/journal.pone.0289082 (PMC10403070; doi:10.1371/journal.pone.0289082)
Supplement: Attachment — Submitted filename: Response to reviewers.docx [file pone.0289082.s005.docx]

The authors would like to appreciate and acknowledge the reviewers, who voluntarily spent their precious time in reading and suggesting changes in order to make this manuscript better. Thank you!

**Response to concerns raised by Reviewer #1.**

After reading the submitted manuscript entitled “In silico and expression analysis shows miR-1183 to be up-regulated in colorectal cancer and targets cell cycle progression gene 1”, we noticed that authors used the available expression data in a good rationale and inferred important conclusions related to their work, however, some additional work is required for this manuscript to be accepted for publication:

1) The manuscript requires English edition

**Response:** We appreciate your attention to detail in enhancing the readability of the manuscript. The manuscript was carefully revised by Mr. Afraz Ahmad to improve the language, sentence structure, and remove grammatical errors.

2) Authors claimed that CCPG1 is a miR-1183 target but didn’t give sufficient bioinformatics and experimental evidences about this relationship. Accordingly, additional work is required to support this suggestion, including: a figure that illustrates the interaction between the miRNA and its target site in the CCPG1 mRNA, in addition to experimental work to determine the impact of ectopic miR-1183 over-expression/down-regulation on the expression of CCPG1 at the mRNA and protein levels. Moreover, Dual Luciferase Assay gives important evidence about this targeting relationship.

**Response.** We thank the reviewer for the valuable comments. No doubt, these experiments will make our claim much stronger. Unfortunately, we do not have the resources and infrastructure to undertake these experiments at this time. We are looking for external collaborators to work in this direction. However, we have now incorporated a figure (4A) depicting the base pair complementarity of miR-1183 seed sequence and miRNA response elements (MRE) in the 3’UTR of *CCPG1* mRNA. Furthermore, we have also changed and updated the title of our manuscript to better align it with our expression data.

**Response to concerns raised by Reviewer #2.**

Fatima et al. proceeded with retrieval of four CRC datasets from GEO and found 2 significant miRNAs (i.e., hsa-miR-1183 and hsa-miR-630) overlapping between all the four CRC datasets. They proceeded with miR-1183 (oncomiR) by identifying its target genes, expression, validation, survival, and PPI analysis. Lastly, they checked for common miRNAs in CRC1, Gastric and liver cancer for establishing metastasis point of view.

1) Colorectal carcinoma (CRC) is among the foremost malignancies with high mortality rate worldwide (Sung et al., 2005)". The reference mentioned in the introduction section is quite old and needs to be updated as per the latest data.

**Response.** The reference has been updated with the new reference. “Colorectal carcinoma is among the foremost malignancies with a high mortality rate worldwide (Rawla et al. 2019)”.

2) Abbreviations should be mentioned in the first place of their occurrence. The authors have used abbreviated terms and full forms somewhere at other places.

**Response.** We have rectified this issue in our manuscript. Specifically, we have eliminated the unnecessary repetition of abbreviated terms, thereby enhancing the readability of our manuscript.

3) The introduction section lacks novelty, strength, and limitations of the study and needs to be explained in detail. A brief paragraph summarizing the study design and results snippets also needs to be incorporated in detail.

**Response.** We have carefully reviewed and incorporated an additional paragraph that emphasizes the novelty, significance, and limitations of our study. This new paragraph provides a comprehensive overview of the study design, highlighting its strengths and addressing the limitations. These additions serve to enhance the overall clarity, depth, and impact of the introductory section, providing readers with a solid foundation for understanding the scope and implications of our study.

4) The abstract section needs to be re-written. Usually the abstract encompasses background, study design, results, conclusions.

**Response.** The abstract was rewritten in line with your valuable comments.

5) There are too many typos and grammatical errors throughout the manuscript and needs to be checked by a native English speaker or a professional English editing software.

**Response.** We appreciate your attention to detail in enhancing the readability of the manuscript. The manuscript was carefully revised by Mr. Afraz Ahmad to improve the language, sentence structure, and remove grammatical errors.

6) The authors have not mentioned why they retrieved gastric cancer and hepatocellular cancer datasets apart from CRC?

**Response.** Previously, solitary and metachronous metastasis of CRC in HCC (Okano et al., 2002; Scheele et al., 1995), HCC into GC (Shirabe et al., 2006) and of gastric cancer into HCC (Garancini et al., 2012) is reported. In this study, for the first time, we identified CRC-related miRNAs and relate it with GC and HCC together.

We have mentioned the above reason for choosing these datasets in discussions. We now have incorporated some additional sentences in section 3.10, first paragraph in order to make it clearer.

7) The datasets inclusion and exclusion criteria must be properly mentioned in the methods section.

**Response.** As advised, the inclusion criteria has now been added in the first paragraph of the methods section

8) The section named "Clinical Information of colorectal cancer (CRC)" in the methods section seems to be irrelevant as it discusses information about CRC datasets. It must be either placed in the supplementary file or as results section.

**Response.** Thank you for pointing out this error. We too believe this section was not well placed. We have now moved this section to the results section (3.1) in line with your suggestion.

9) "R-Bioconductor package was used for data normalization". Please mention which package? The authors have not mentioned any information regarding datasets pre-processing which usually involves normalization, log2 transformation, batch correction, gene mapping, etc. The information currently mentioned seems misleading to reader.

**Response.** Thank you for pointing out the confusion. We have updated the methods section with the explanation regarding sample preprocessing, normalization and differential expression. However, the batch correction was not performed in the current analysis as it was required for the cohorts under study. The modifications can be seen in section 2.1 and 2.2 of the manuscript.

10) The authors used only TargetScan for their target search? Why. Please justify. There are other validated sources available also such as miRWalk, miRDB, miRTarBase, etc.

**Response.** Initially, we used TargetScan for the target search of miRNA. Following this suggestion, we included miRmap in addition to TargetScan in our manuscript. In addition to miRmap and TargetScan, the target of miR-1183- CCPG1, is also present on miRWalk.

11) It would be nice if the mRNA dataset of CRC also comprised same patients in CRC1/CRC2/CRC3/CRC4 as it would justify the results properly.

**Response.** Yes, we agree with the worthy comment. CRC1 dataset in our study includes expression of both miRNA and mRNA of the same patients.

12) The KM curve shows a nonsignificant log rank p-value (p-value = 0.33). How can CPG1 is prognostically significant?

**Response.** You have rightfully pointed out the error. After careful consideration and review, the decision was made to remove the KM curve from the manuscript. By removing the KM curve, we aimed to enhance the overall professionalism and effectiveness of the manuscript, providing a more cohesive and impactful narrative.

13) What cutoff did the authors chose for constructing PPI network from STRING? Please mention.

**Response.** In the construction of the protein-protein interaction (PPI) network, a cutoff value of 0.4 was selected based on careful consideration of various cutoff thresholds such as 0.15 (low), 0.40 (medium), 0.70 (high), and 0.90 (highest). We have also incorporated it in our manuscript (section 3.9 of the manuscript).

14) The quality of figures needs to be improved throughout the manuscript.

**Response.** The figures within our manuscript have been thoughtfully revised and adjusted to align with the prescribed formatting standards outlined by PLOS One. The high-quality figures are now uploaded as separate files.
